# Optimizing data collection for public health decisions: a data mining approach

**DOI:** 10.1186/1471-2458-14-593

**Published:** 2014-06-12

**Authors:** Susan N Partington, Vasil Papakroni, Tim Menzies

**Affiliations:** 1 Division of Animal and Nutritional Sciences, West Virginia University, Morgantown, WV, USA; 2 Lane Department of Computer Sciences and Electrical Engineering, West Virginia University, Morgantown, WV, USA; 3 Regional Research Institute, West Virginia University, 886 Chestnut Ridge Road, 5th Floor, P.O. Box 6825, Morgantown, WV 26506-6825, USA

**Keywords:** Community survey methods, Data mining, Data collection, Ecological and environmental concepts, Nutrition

## Abstract

**Background:**

Collecting data can be cumbersome and expensive. Lack of relevant, accurate and timely data for research to inform policy may negatively impact public health. The aim of this study was to test if the careful removal of items from two community nutrition surveys guided by a data mining technique called *feature selection*, can (a) identify a reduced dataset, while (b) not damaging the signal inside that data.

**Methods:**

The Nutrition Environment Measures Surveys for stores (NEMS-S) and restaurants (NEMS-R) were completed on 885 retail food outlets in two counties in West Virginia between May and November of 2011. A reduced dataset was identified for each outlet type using feature selection. Coefficients from linear regression modeling were used to weight items in the reduced datasets. Weighted item values were summed with the error term to compute reduced item survey scores. Scores produced by the full survey were compared to the reduced item scores using a Wilcoxon rank-sum test.

**Results:**

Feature selection identified 9 store and 16 restaurant survey items as significant predictors of the score produced from the full survey. The linear regression models built from the reduced feature sets had R^2^ values of 92% and 94% for restaurant and grocery store data, respectively.

**Conclusions:**

While there are many potentially important variables in any domain, the most useful set may only be a small subset. The use of feature selection in the initial phase of data collection to identify the most influential variables may be a useful tool to greatly reduce the amount of data needed thereby reducing cost.

## Introduction

Ideally, public health policy should be informed by research, assessments and surveillance [1]. These activities rely on the availability of current and accurate data collected at the both the individual- and community-levels [2]. The cost of conducting health research has recently become an important consideration due decreases in available funding. In the United States, federal funding for biomedical research as a percent of total health care expenditures decreased from 11% to 2% from 1980 to 2010 [3].

This paper explores one approach for reducing research costs by reducing the number of survey items on two community nutrition assessment instruments. In principle, the approach described here is quite general and could be applied to reducing the amount of data needed to assess outcomes across a wide variety of health research questions.

## Background

Collection of primary data is one of the most expensive and time consuming aspects of any research study [4]. To ensure data integrity, the collection process must be consistently monitored. After collection, information from paper forms requires double entry by hand or machine scanning followed by manual confirmation of scanner accuracy. Electronic collection of data either in person or over the internet requires the purchase or development of software to collect the data and if deployed over the internet, web-based tools and the resources to host them [5]. In all cases, data cleaning and validation is required [6]. Resources needed increase in proportion to the amount of data to be collected and managed. Further even a rigorously monitored data gathering process is error prone. Transcription errors, recording errors, data entry errors, and errors resulting from equipment malfunction all have the potential to distort findings and compromise results [7]. Minimizing the amount of data needed to produce an accurate assessment minimizes research costs as well as the risk of errors.

### Data mining

Data mining techniques employ algorithms or learners that can build prediction models. Such algorithms include linear regression, decision tree learners, Bayes classifiers, random forests and support vector machines among others [8]. Within these learners, there is often a feature selection algorithm that identifies elements within a dataset that are useful in the prediction model. There are many feature selection algorithms including stepwise regression, principle component analysis [9] and information gain [8]. Feature selection studies have found that ranking of singleton variables (as in stepwise regression) does not work as well as exploring the rankings of combinations of variables. That is, if every variable were ranked only by their independent association to the outcome, important pairs (or triples or higher-order) of influences would be missed. Principle components analysis is useful for data reduction but does not consider an outcome [10]. While detection of highly influential variables can inform data reduction strategies, if there is an outcome of interest, the association of variables with this measure is equally important. Feature subset selection algorithms can explore an exponential number of combinations of variables. Hence, the resulting selection is based on influences among independent variables as well as their association to the outcome.

A comparative study by Halls and Holmes reviewed numerous feature selection algorithms among them Hall’s Correlation-Based Feature Selection (CFS) algorithm [10]. CFS produces a subset of variables or reduced dataset without losing the essential signal in the data [10,11] by identifying variables that are strongly associated to the target class (outcome) and weakly associated with each other. The most useful feature subset is then identified using a best first search. Best first is an iterative algorithm that keeps track of the current best subset of variables and candidate subsets not considered yet. Best first makes guesses in order to achieve some goal. In most practical applications, there are too many guesses to explore. To cull that very large space, best first search conducts an initial exploration, then sorts the best guesses produced so far. It then explores further, but only in the sorted order (to sort the current subset of features, CFS uses its internal entropy measurements). On every iteration, the algorithm picks the most promising subset and compares its predictive value against that of the best subset identified thus far. The search terminates when, after a fixed number of trials, no subset is found to significantly improve the prediction. To test the external validity of the selected features rigorous a cross-validation procedure is used where some data is kept in reserve and the model tested using the hold-out set.

### Surveys

The Nutrition Environment Measures Surveys for Stores (NEMS-S) and Restaurants (NEMS-R) are audit tools used to assess the community nutrition environment [12,13]. NEMS-S reports the availability and cost of healthy options compared to less healthy options in retail food outlets over 9 food groups as well as the availability, cost, and quality of fresh fruits and vegetables [13]. The Nutrition Environment Measures Survey for restaurants (NEMS-R) measures eight aspects of the nutrition environment including: availability, access, nutrition information, price, nutrition quality, and barriers [12]. A sum score is produced from survey data. The possible score for stores ranges from -9 to 54, the restaurant score from -27 to 63 [14,15]. Higher scores relative to lower scores infer greater availability of and lower cost of healthy options, as well as support for healthy choices. Testing of psychometric properties (reliability, validity) has been completed. Both surveys exhibited high inter-rater and test-retest reliability and construct validity [16]. Although the NEMS surveys produce reliable and valid assessments and are usable in a wide variety of outlets and communities, the effort associated with their administration is a major limitation to their use.

## Methods

### Data collection

Using the NEMS surveys, retail food outlets in two West Virginia counties were audited between May and November, 2011 as part of the West Virginia Early Childhood Obesity Prevention Project. Outlets were enumerated using a list of all businesses in the two counties and 5 mile buffer with primary or secondary Standard Industrial Classification (SIC) codes beginning in 53 (retail-department, variety, and general merchandise stores), 54 (retail-food stores), and 58 (retail-eating and drinking places) purchased from InfoUSA in February 2011. Establishments were identified as supermarkets, grocery stores, convenience stores and variety, general merchandise or department stores. Restaurants as sit-down, fast casual, fast food and specialty. A trained auditor visited each outlet and completed the survey using a paper form.

### Data processing

Completed survey forms were scanned and data verified using TeleForm version 10.6. Survey scores were computed from the raw data according to instructions [14,15]. Scoring for NEMS-S assigns point values ranging from 0 to 3 to the availability of healthier items in 11 food groups: milk, fruits, vegetables, ground beef, hot dogs, frozen dinners, baked goods, beverages, chips and cereal [15]. The cost of healthier items compared to similar less healthy items in the same food groups (except fruit and vegetables) are assigned a point value of -1 if the cost of the less healthy option is less and 2 points if the healthier is less costly [15]. Quality of fruits and vegetables is assigned a point value of 1–3 based on the percent of the 10 fruits and 10 vegetables included on the survey that are acceptable in quality [15]. NEMS-R scoring assigns 3 points to the availability of healthy entrees and salads, whole grain bread, baked chips, 100% fruit juice and 1% or low-fat milk [14]. Two or three points are assigned to facilitators and supports of healthy eating such as nutritional information and reduced-size. Barriers to healthy eating are present such as all-you-can-eat format portions are assigned -3 points [14]. Assigned values are summed to produce a score for each outlet type.

The full surveys included numerous alternate and conditional responses. This expanded the number of columns in the resulting dataset. For example, on the NEMS-S the availability of whole wheat bread is queried three times (two specific brands and any alternate). Alternate item responses and pricing were combined to produce single variables when possible. Similarly, the NEMS-R included 23 questions related to restaurant hours of operation. These were combined to produce four variables that represented total hours of operation on the four days queried.

### Data reduction

The Waikato Environment for Knowledge Analysis (WEKA), version 3.6.8. was used for implementation of CFS, linear regression and cross-validation. Store and restaurant datasets were processed separately with NEMS score as the class or dependent variable. The CFS feature selector uses the best first search procedure (described above). CFS ranks variables according to how often they are included in the best subset or the group of variables that minimizes the error rate of the predictive model [8]. To ensure the features selected will be applicable to additional (new) survey data, evaluation was completed using a cross-validation procedure instead of testing the model on the same data set used to develop it. Ideally different datasets would be used to find the best subset and one to test [8]. In this study cross-validation was used due to the limited number of food outlets in the data set. WEKA executes a 10 fold cross-validation procedure that randomly divides the data into 10 parts or folds, each tenth of the data is used to calculate the error rate for the subset selected using the other 9 parts [8].

Finally, working with the attributes selected by CFS, WEKA performs a standard ordinary least squares multiple linear regression using Akaike Information Criterion (AIC) as a termination criteria to determine attributes to be retained in the final model [8]. The (AIC) is computed as the negative log likelihood plus the number of parameters in the model [8]. It is an estimate of model fit with a penalty for complexity [8]. All attributes entered into the model are eliminated based on decreasing value of the standardized coefficient [8]. The elimination process stops when the AIC score is minimized, that is, when the AIC is not improved by further attribute elimination [8]. Models were constructed using the same 10 fold cross-validation procedure.

The final regression models included variables that were significant in predicting NEMS scores at alpha < = .05. Values of items in the reduced models for stores and restaurants were weighted by the regression coefficient and summed with the constant to produce the reduced item scores. Median values of reduced item and the full survey scores were compared for all stores and restaurants and for outlet type within these two categories using a Wilcoxon signed-rank test and score agreement assessed using the concordance correlation coefficient (Table 1). Stata version 12.0 was used for computing NEMS survey scores, reduced item scores, model coefficient standard errors and for comparison of the full NEMS survey and reduced item scores.

**Table 1:** Comparison of full NEMS surveys and reduced item scores by outlet type

**Outlet type**	***n***	**Full survey score median (IQR)**	**Reduced survey score median (IQR)**	***p***^**1**^	**Score agreement**^**2 **^**(95% CI**^**3**^**)**
All stores	301	10.0 (7.0, 15.0)	10.3 (7.0, 16.2)	0.818	0.926 (0.909 - 0.941)
Grocery	66	25.0 (17.0, 27.0)	24.0 (17.5, 26.0)	0.389	0.838 (0.755 - 0.894)
Convenience	138	10.0 (7.0, 12.0)	10.0 (7.0, 11.7)	0.671	0.860 (0.810 - 0.898)
Variety^4^	97	8.0 (6.0, 12.0)	8.4 (6.6, 10.3)	0.543	0.846 (0.786 - 0.891)
All restaurants	584	8.0 (2.0, 17.0)	6.8 (0.7, 16.2)	0.148	0.925 (0.912 - 0.935)
Sit-down	218	7.5 (3.0, 14.0)	6.1 (1.3, 13.3)	0.119	0.905 (0.878 - 0.926)
Fast casual	111	5.0 (0.0, 10.0)	4.6 (0.8, 10.5)	0.184	0.871 (0.821 - 0.907)
Fast foods	242	9.0 (3.0, 22.0)	9.3 (3.0, 19.2)	0.082	0.939 (0.922 - 0.952)
Specialty	13	6.0 (0.0, 9.0)	3.5 (0.7, 6.9)	1.000	0.890 (0.700 - 0.962)

^1^Wilcoxon signed-rank test, 2-sided.

^2^Concordance correlation coefficient.

^3^Fisher’s z-transformed CI.

^4^Includes department, dollar and general merchandise stores.

## Results

Audits were completed on 301 stores and 584 restaurants in the two counties. After text fields were eliminated, the NEMS store and restaurant data sets contained 351 and 93 variables respectively. After combining items related to the availability and price of the same food and hours of operation the NEMS-R data set contained 74 variables and NEMS-S 112. The feature selection process identified 9 survey items that predicted the full NEMS-S score and 16 that predicted NEMS-R. When the selected items were entered into linear regression models, the R^2^ statistic was 0.921 for NEMS-S and 0.936 for NEMS-R (Table 2). The median scores produced by the sum of the weighted reduced items were similar to the full survey scores over all outlet types. The signed-rank test showed no statistically significant differences in the median scores (Table 1). The CCC values at 0.926 for stores and 0.925 for restaurants indicated good agreement between full NEMS survey scores and the reduced item scores. CCC values produced for all sub-types of stores and restaurants ranged between 0.838 and 0.939 and also indicated good agreement between survey versions.

**Table 2:** Data mining results, linear regression, class (dependent) variable: total NEMS score

**Stores: NEMS-S survey item**	**Item response**	**Value**	**Coefficient (SE)**
Banana quality	Acceptable, not acceptable/available	1, 0	3.477 (0.641)
Apples available	yes, no	1, 0	2.513 (0.640)
Carrots available	yes, no	1, 0	1.657 (0.969)
Lettuce available	yes, no	1, 0	3.650 (0.963)
Fat free hotdogs available - any brand	yes, no	1, 0	6.501 (0.607)
Baked chips available – any brand	yes, no	1, 0	3.263 (0.357)
Whole wheat bread available - any brand	yes, no	1, 0	1.368 (0.367)
Skim or low-fat milk available (any size)	yes, no	1, 0	1.725 (0.482)
Half gallon milk price: skim or low-fat compared to whole	regular > skim/lowfat, regular < skim/lowfat	1, 0	1.966 (0.347)
Correlation coefficient = 0.921	constant		3.322 (0.502)
**Restaurants: NEMS-R survey item**	**Item response**	**Value**	**Coefficient (SE)**
Nutrition information available on-site	yes, no	1, 0	3.901 (0.614)
Identification of healthier menu items on take-away menu	yes, no	1, 0	3.617 (0.620)
Identification of healthier menu items on website menu	yes, no	1, 0	3.445 (0.597)
Nutrition information posted near POP^1^ or available in brochure	yes, no	1, 0	2.997 (0.635)
Signs, table tents, displays highlight healthy menu options	yes, no	1, 0	2.450 (0.790)
Signs, table tents, displays encourage healthy eating	yes, no	1, 0	3.243 (0.832)
Baked chips available	yes, no	1, 0	2.756 (0.620)
100% wheat or whole grain bread available	yes, no	1, 0	4.735 (0.497)
100% fruit juice available	yes, no	1, 0	2.496 (0.481)
1% low-fat, skim, or non-fat milk available	yes, no	1, 0	2.109 (0.529)
Fruit (w/out added sugar) available	yes, no	1, 0	3.368 (0.493)
Non-fried vegetables (w/out added sauce)	yes, no	1, 0	4.071 (0.396)
Main dish salads: healthy options available	yes, no	1, 0	2.614 (0.417)
Main dishes/entrees: healthy options available	yes, no	1, 0	2.763 (0.476)
Healthy entrees identified on menu	yes, no	1, 0	3.177 (0.534)
All-you-can-eat or “unlimited trips”	yes, no	1, 0	-4.882 (0.560)
Correlation coefficient = 0.936	constant		1.034 (0.280)

^1^Point of purchase.

## Discussion

The goal of this study was to find the most influential survey items in predicting the total survey scores. The NEMS scores are numeric making CFS and linear regression good approaches. CFS is also a very fast feature selector; hence it is recommended when processing data sets with hundreds of attributes (such as the NEMS data). However, there are a variety of techniques and tools available, the optimal technique is dependent on the type of data, how much data is available, intended use and purpose of the data reduction [17]. Nominal or ordinal outcomes would require alternative methods. For example, if all the attributes had discrete value then it can be more insightful to apply association rule learning [18] and report those parts of that model with most confidence and support. Also, for data sets with just a few rows or attributes, the wrapper feature selector [11] might produce better results since this slower feature selector checks each candidate subset by calling out a learner to generate a model for that subset.

The data mining process provided a means to identify the most influential data points in predicting total survey scores and eliminate redundancies resulting in a survey with many fewer required responses and weighted scores comparable to the original. The CCC is a measure of agreement developed to assess reproducibility [19]. Coefficients in the range of 07 to 1.0 indicate good agreement, 0.4 to 0.7 moderate agreement and less than 0.4 poor agreement [20]. According to this scale, agreement between the scores produced by the full surveys and those from the reduced dataset was good for both stores and restaurants and for all sub-types of these two larger categories.

Although the reduced item surveys have not been field tested, it is likely that the time required to complete an audit will be decreased. Collection of a smaller number of survey items will also reduce time needed for data entry and processing and the complexity of computing scores. Even though the reduced datasets had high correlations to the full survey scores, there was some degree of information loss. Specifically, the extensive food item detail contained in the full versions has been eliminated. While essential for micro-level examination of the food environment, when there are a large number of outlets to be surveyed or repeated measurement is required for surveillance, a reduced item survey provides a feasible method to produce total survey scores.

## Conclusions

While this study used survey construction as an example, feature selection may be a very useful tool to reduce data collection burden by guiding ongoing data collection. For example, feature selection could be applied to an initial dataset and the scope of subsequent data collection narrowed by identifying and eliminating redundant features that are redundant in predicting the outcome of interest. Given current levels of research funding, it seems prudent to explore alternate and potentially cost saving methods.

## Abbreviations

NEMS-S: Nutrition Environment Measures Survey - Stores; NEMS-R: Nutrition Environment Measures Survey – Restaurants; WEKA: Waikato Environment for Knowledge Analysis; WV: West Virginia; SIC: Standard Industrial Classification; CFS: Correlation-based Feature Selection; AIC: Akaike Information Criterion.

## Competing interests

The authors declare that they have no competing interests.

## Authors’ contributions

SP conceived of and designed the study, performed the statistical analysis, and participated in drafting the manuscript. TM directed the data mining and participated in drafting the manuscript. VP performed the data mining procedures. All authors read and approved the final manuscript.

## Pre-publication history

The pre-publication history for this paper can be accessed here:

http://www.biomedcentral.com/1471-2458/14/593/prepub
